# Reassessing Viral Origins: From Escaped Genes to Degenerated Microbes

**DOI:** 10.3390/pathogens14121205

**Published:** 2025-11-26

**Authors:** Peter Borger

**Affiliations:** The Independent Research Initiative on Information & Origins (TIRIO), Wort und Wissen, 72250 Freudenstadt, Germany; peterborger@hotmail.com

**Keywords:** virus, evolution, exogenization, endogenization

## Abstract

Three main hypotheses have been proposed to explain the origin of viruses: the exogenisation (escape) hypothesis, suggesting that mobile genetic elements gained infectivity and autonomy; the degeneration hypothesis, proposing that viruses arose through gene loss from more complex, possibly cellular ancestors; and the virus-first hypothesis, which argues that viruses are ancient, pre-cellular entities. This review evaluates these models in light of molecular, structural, and ecological evidence. Key considerations include the lack of homologues for many viral proteins, the presence of giant DNA viruses with extensive gene repertoires, the conservation of capsid structures across diverse viruses, and the universal dependence of viruses on living hosts. Also discussed is the vast diversity of the global virosphere revealed by recent metaviromic studies, particularly in marine ecosystems, where viruses play key roles in structuring microbial communities and driving biogeochemical cycles. Such findings highlight that viruses are integral components of biological systems rather than merely parasitic outliers. Although no single hypothesis fully explains the origin of all viruses, their extraordinary genetic and functional complexity suggests a unified evolutionary theory may forever remain elusive. Rather, understanding the origins of viruses requires integrating genomic traits with ecological roles across their wide diversity.

## 1. Introduction

The origin of viruses remains one of the most intriguing and unresolved questions in evolutionary biology. Despite major advances in comparative genomics, structural virology, and metagenomics, there is still no consensus on whether viruses originated before or after the emergence of cellular life. Their profound dependence on host organisms for replication, combined with their immense genetic diversity and the absence of universally conserved genes, has led to the development of multiple origin models. These include the virus-first hypothesis, which posits that viruses predate cellular life; the escape (or “exogenization”) hypothesis, suggesting that viruses emerged from mobile genetic elements that acquired the ability to move between cells; and the reduction (or degeneration) hypothesis, which sees viruses as the remnants of once-independent cellular organisms that gradually lost their autonomy.

Among these, the exogenization and microbe-degeneration models continue to receive considerable attention and refinement, especially as new large DNA viruses blur the distinction between viruses and cellular organisms. The discovery of nucleocytoplasmic large DNA viruses, such as mimiviruses and pandoraviruses, has revived interest in the idea that some viral lineages may have descended from ancestral cells that underwent genome reduction, shedding genes no longer needed in a host-dependent lifestyle [1,2]. Experimental evidence shows that genome size and gene content in giant viruses can shrink under certain conditions, supporting the degeneration hypothesis [3]. At the same time, the existence of autonomous mobile genetic elements—such as plasmids [4,5], transposons and retroelements [6]—that share functional features with viruses has lent support to models suggesting that viruses evolved through exogenization processes: the outward escape of genomic material from host cells, eventually forming infectious agents capable of intercellular transfer [7,8].

These models are not mutually exclusive and may reflect different origins for different classes of viruses. For example, RNA viruses may have followed an entirely distinct trajectory from DNA viruses, and even within DNA viruses, distinct groups could have emerged from separate ancestral events. In this context, some recent theoretical work has proposed variations on these classical hypotheses reviewed in [9]. These perspectives suggest that the origin of viruses might be intimately tied to the functional regulation of mobile genetic elements within cellular genomes, and that these elements may have been exogenized under specific conditions, potentially as part of adaptive mechanisms. The microbe-degeneration theory, on the other hand, is often framed not only in terms of genome reduction but also as a process of functional de-specialization from symbiotic or parasitic ancestors.

This paper aims to evaluate the explanatory power and limitations of these two competing models—exogenization and microbe-degeneration—within the context of current virological knowledge. By considering genomic, structural, and molecular evidence, we aim to assess how well each theory accounts for the key features of viral biology, including genome composition, replication strategies, and phylogenetic distribution. Special attention is given to how the role of transposable elements and endogenous viral elements may inform our understanding of viral origins. The frequent integration of viral sequences into host genomes, as well as their regulation via epigenetic mechanisms, highlights the highly dynamic relationships between viruses and their hosts [10,11]. In doing so, we seek to clarify whether the virus–host relationship is best seen as the result of genetic escape, degenerative evolution, or a more complex interplay of both processes.

### 1.1. Established Theories on the Origin of Viruses

A variety of hypotheses have been proposed to explain the origin of viruses, with the three most widely discussed being the escape hypothesis (also called the exogenization hypothesis), the reduction or degeneration hypothesis, and the virus-first hypothesis. Each model attempts to reconcile the unique biological features of viruses—such as their dependence on host cells for replication and their diverse genomic architectures—with comparative genomic and phylogenetic data.

### 1.2. Escape (Exogenization) Hypothesis

The escape hypothesis posits that viruses originated from autonomous genetic elements—such as plasmids, transposons, or retrotransposons—that acquired the ability to exit the cell, encapsulate themselves in protein coats, and move between host cells. Over time, these mobile genetic elements would have evolved increasing levels of complexity, acquiring genes necessary for infection, replication, and evasion of host defenses. This model is supported by the existence of transposable elements that resemble viral genomes in structure and function, and by the well-documented capacity of viruses to capture host genes [8,12].

One of the major strengths of the exogenization model lies in its ability to explain the mosaic nature of many viral genomes, which frequently contain host-derived genes and show signatures of recombination with other genetic elements. Additionally, the structural and functional similarities between some viral proteins and those involved in plasmid conjugation and transposition further support a common origin [5]. Supporting this view, recent findings have shown that certain retroviruses share functional vulnerabilities with endogenous retroviral elements, as their activity can be effectively inhibited by the same reverse transcriptase-targeting drugs, such as zidovudine and efavirenz, suggesting a shared mechanistic basis and possibly a common origin from mobile genetic elements within the host genome [13].

However, the escape hypothesis struggles to fully account for the origin of hallmark viral features such as capsid proteins and dedicated replication machinery, many of which lack close homologs in cellular life. The de novo emergence of such components remains speculative, and the phylogenetic disconnection of many viral hallmark genes from known cellular lineages limits the explanatory power of this model for certain virus groups [14,15]. Nonetheless, the escape hypothesis is strongly supported by molecular genetic evidence, as illustrated by the following example.

### 1.3. The Origin of Rous Sarcoma Virus (RSV)

RNA viruses contain compact yet highly functional genetic elements that enable them to replicate as molecular parasites. Typically, an RNA virus possesses only a limited number of genes. This raises a key question: where did these genes originate? The most parsimonious explanation is that RNA viruses escaped from genomic elements that already carried these genes, eliminating the need for their de novo origin. Endogenous retroviruses (ERVs)—recently reidentified as regulatory genetic elements—may represent the source of many retroviruses. These ancient genomic elements harbor gene architectures—gag (group-specific antigen, encoding structural proteins that form the capsid and matrix), pol (polymerase, encoding enzymes essential for viral replication, including reverse transcriptase, integrase, and protease), and env (envelope, encoding glycoproteins that mediate host cell recognition and entry)—that are virtually identical to those found in modern retroviruses, suggesting a direct lineage [16].

**Figure 1:**
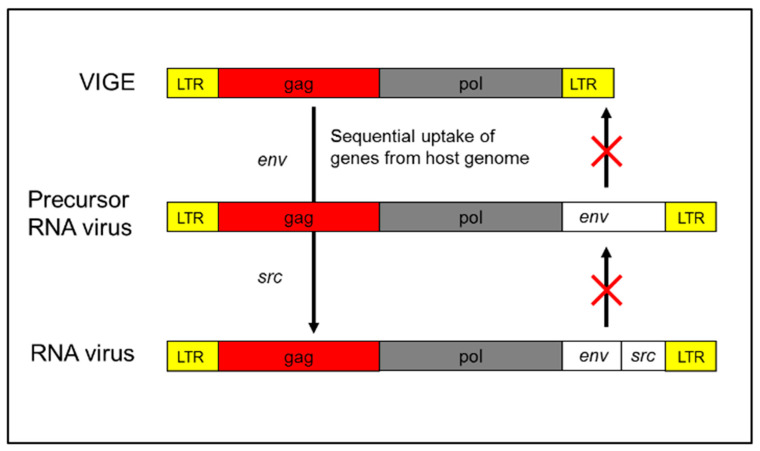
Origin of Rous Sarcoma Virus (RSV). The Rous Sarcoma Virus (RSV) is hypothesized to have originated from an ancestral ERV through a two-step molecular genetic process. First, the acquisition of an envelope (env) gene enabled the element to form a functional virion, resembling members of the HERV-K family—human-specific endogenous retroviruses. Subsequently, a recombination event with host genomic material led to the incorporation of a portion of the SRC proto-oncogene, conferring oncogenic potential. Once these modifications were complete, the element emerged as an infectious retrovirus with the capacity for unregulated replication and horizontal transmission. This model highlights the dynamic role of ERVs as both genomic elements and potential reservoirs for the emergence of pathogenic retroviruses.

A compelling case is provided by Rous Sarcoma Virus (RSV), an oncogenic retrovirus that harbors only four genes: gag, pol, env, and src. Flanking these coding regions are long terminal repeats (LTRs), which promote integration into the host genome and enhance viral replication. The gag, pol, and env genes are canonical retroviral genes and are frequently found in ERVs. The src gene, however, is of particular interest—it is a modified version of a host-derived src gene, originally encoding a tyrosine kinase involved in normal cell signaling. In RSV, this gene has been altered to function as a constitutive activator of cell proliferation, effectively converting it into an oncogene. Importantly, src is not essential for viral replication. RSV variants lacking src—retaining only gag, pol, and env—remain replication-competent but lack oncogenic potential [17]. This strongly supports the view that the src gene was captured from the host genome (Figure 1). Given this, it is reasonable to hypothesize that the entire viral vector may have originated from host genetic material.

ERVs are particularly prone to acquiring host-derived sequences through mechanisms such as RNA polymerase II read-through transcription or incomplete excision events. Thus, many RNA viruses may have assembled from preexisting host sequences. For example, the outer envelope of the influenza virus is composed of hemagglutinin and neuraminidase—proteins with clear homologs in higher eukaryotes. Neuraminidase, in particular, plays a critical role in glycopeptide and oligosaccharide processing in humans. Mutations or deficiencies in this enzyme can lead to severe lysosomal storage disorders. Even so-called “orphan genes”—those with no known homologs in cellular life—are often found embedded within ERV loci in host genomes. This suggests that ERVs may serve as genetic reservoirs or intermediates in the origin of novel viral genes [18].

These findings support the notion that RNA viruses likely originated through recombination events involving host genomic elements such as protein-coding genes, promoters, and enhancers—often mediated or facilitated by ERV activity. Occasionally, an “unfortunate” recombination event involving an ERV may produce a self-replicating molecular unit, marking the genesis of a novel viral particle. Once such a construct acquires the ability to re-enter host cells, it transitions into a fully infectious virus. It has long been recognized that bacteria can acquire advantageous traits from bacteriophages—viruses that integrate into the bacterial genome either transiently or permanently. In many cases, prophage genes are responsible for encoding major bacterial toxins. In such scenarios, viral genes confer reproductive advantages upon their bacterial hosts in specific environments. The emergence of such pathogenic viruses is thus best understood not as a purely linear descent, but as a dynamic process of genetic recombination and mosaic assembly, involving existing mobile elements such as plasmids, insertion sequences, and endogenous retroviruses. Rather than emerging de novo, many viruses appear to be recombinants of host-derived genetic modules, repurposed for parasitic replication [6,19].

### 1.4. Reduction (Degeneration) Hypothesis

The reduction hypothesis suggests that viruses originated from more complex, possibly cellular ancestors—such as parasitic or symbiotic microorganisms—that gradually lost genes no longer essential to a host-dependent lifestyle. This model is particularly supported by the discovery of giant viruses, such as mimiviruses and pandoraviruses, which possess large genomes encoding components typically associated with cellular life, including DNA repair, translation-related functions, and nucleotide metabolism [1,2,20]. These findings imply that such viruses may be remnants of once-free-living or parasitic cells that underwent reductive evolution [7,21].

### 1.5. Evidence for the Reduction Hypothesis

In 2003, the discovery of Mimivirus challenged conventional views on viral complexity. Mimivirus is an exceptionally large virus, approximately 750 nanometers in diameter, visible under light microscopy, and infects amoebae. Its genome, comprising 1.2 million base pairs and over 900 genes, rivals the size of some bacterial genomes [22]. Remarkably, Mimivirus encodes genes typically absent in viruses, such as those for tRNA synthetases—enzymes crucial for protein synthesis—and three types of topoisomerases (IA, IB, IIA) involved in DNA topology regulation during replication. These topoisomerase genes are homologous to those found in soil bacteria, suggesting a bacterial ancestry [22]. The presence of such genes implies that Mimivirus likely evolved from a free-living bacterium that progressively lost non-essential genes while adapting to an obligate parasitic lifestyle within amoebae [23,24]. This evidence strongly supports the reduction hypothesis, positing that certain viruses derive from degenerate cellular ancestors that gradually lost autonomy.

More recently, in 2025, researchers identified *Sukunaarchaeum*, a novel microorganism detected incidentally during the genomic analysis of the marine dinoflagellate *Citharistes regius*. *Sukunaarchaeum* possesses a remarkably small circular genome of only 238,000 base pairs encoding 189 genes, fewer than many viruses [25]. Most genes facilitate self-replication, but the organism lacks key genes for energy metabolism and biosynthesis, indicating an obligate dependence on its host. This genomic minimalism, coupled with its virus-like lifestyle, suggests that *Sukunaarchaeum* is a degenerate archaeon undergoing reductive evolution, transitioning toward a virus-like existence [25].

Together, these findings from Mimivirus and *Sukunaarchaeum* reinforce the concept that viruses may originate from cellular organisms undergoing progressive gene loss and functional simplification. Rather than representing a primitive or early form of life, viruses might be the products of evolutionary regression from once free-living cells. This challenges traditional paradigms that view viruses solely as emergent biological entities and highlights their role as indicators of cellular degradation and genomic reduction. The reduction hypothesis effectively explains the presence of complex replication systems and large coding capacity in some viruses, aligning with the general evolutionary trend of genome reduction observed in many obligate intracellular parasites and symbionts. Moreover, experimental evidence has demonstrated that viral genomes can undergo rapid reduction when specific functions become redundant due to host dependence [3]. Nevertheless, the reduction model faces challenges as well. The hypothesized transitional forms between cellular organisms and modern viruses have not been clearly identified in extant life, and the loss of genes does not inherently explain the origin of unique viral features, such as capsid formation and highly efficient genome packaging systems. Furthermore, genome reduction is generally a process that optimizes existing parasitism rather than generating novel infectious mechanisms from scratch [26,27].

### 1.6. Virus-First Hypothesis

The virus-first hypothesis proposes that viruses predate cellular life and are remnants of a pre-cellular “RNA world” or other ancient replicator systems. In this model, viruses or virus-like genetic elements were already replicating independently in the primordial environment and later became obligate intracellular parasites following the emergence of the first cellular organisms [28,29].

This hypothesis offers a coherent framework for explaining why numerous viral genes—particularly those encoding capsid proteins and replication enzymes—lack detectable homologs in cellular genomes and appear to be uniquely restricted to the viral world. The structural conservation of viral capsids across highly divergent viral families, despite their lack of sequence similarity, may indicate that some viral components are deeply rooted in primordial biological systems [30,31].

However, the virus-first model is also highly speculative, as all known viruses are obligate parasites that require host machinery for replication. No current virus is capable of independent existence or metabolism, making it difficult to infer how ancestral virus-like entities might have sustained themselves prior to the origin of cellular life [32]. Moreover, the transition from hypothetical pre-cellular replicators to fully parasitic viruses remains not only poorly understood, but no plausible hypothesis has yet been formulated to account for how such a transition could have occurred [33].

## 2. Evaluating the Hypotheses in Light of Empirical Data

Any credible theory of viral origin must account for a set of empirical observations derived from comparative genomics, structural biology, and metagenomics. Two competing models—exogenization (escape) and microbe-degeneration (reduction)—differ in how well they explain such observations. Below I discuss several key data points and how each model fares, referencing recent literature.

Many viral proteins lack detectable cellular homologues. Comparative genomic analyses show that a large fraction of viral genes are “ORFans,” i.e., open reading frames with no discernible similarity to known cellular proteins; in prokaryotic viruses roughly 80 %, and in eukaryotic viruses around 65 %, fall into this category. This suggests a substantial amount of viral genetic novelty [34]. Under the exogenization model, this observation is challenging: while mobile genetic elements might supply some novel sequences, the de novo origin of completely novel protein folds (such as many capsid proteins or replication enzymes) remains difficult to explain completely. The degeneration hypothesis can explain loss of genes in formerly more complex genomes but still must account for the emergence of entirely novel structures (capsids, etc.) that apparently do not derive from known cellular homologues [31,35].

Large DNA viruses with large gene repertoires—so-called giant viruses—present another vital datum of huge diversity [36,37]. Viruses such as Mimivirus and Pandoravirus have genomes larger than those of many bacteria (exceeding 1 Mbp), encoding hundreds of proteins including metabolic, translation-related and other auxiliary functions that are typically associated with cellular life [35]. In the exogenization framework, such viruses must have acquired many genes via gene capture from hosts or mobile elements; in contrast, the degeneration view holds that these viruses descend from more complex cellular ancestors that gradually lost genes but retained many formerly core cellular functions.

Conserved capsid architectures across divergent viral lineages provide important structural constraints. Studies of major capsid protein (CP) folds show that many viruses share capsid architectures, such as single or double “jelly-roll” folds, or HK97 fold, even when their amino acid sequence similarity is minimal or absent [31,38]. Under exogenization, such conservation implies either very early emergence of these capsid folds, or frequent horizontal transfer; the degeneration hypothesis more naturally accommodates the retention of capsid features if the ancestor already possessed them and structural constraints maintain stability.

The absolute dependence of viruses on living host cells for replication is shared by both models: neither escape nor degeneration can deny this core feature. It remains problematic primarily for the virus-first hypothesis, which proposes an origin independent of fully developed cells. Both exogenization and degeneration accept parasitism or dependency as a central feature, though they diverge in the source of the ancestral entities.

Finally, the diversity of RNA vs. DNA viruses must be addressed. RNA viruses tend to exhibit higher mutation rates, smaller genomes, and more rapid adaptive dynamics, whereas DNA viruses often have larger, more complex genomes, sometimes encoding auxiliary metabolic genes and more elaborate replication machinery [22,35]. The exogenization hypothesis might posit that pathogenic RNA viruses may be best explained as originating from mobile RNA elements, while DNA viruses may derive in large part through exogenization of huge DNA elements or through degeneration of former cellular lineages, including bacteria. The degeneration theory can explain why some viruses retain DNA-based replication machinery and greater complexity, but it must also explain how RNA viruses arose with their distinct replication strategies and why many lineages show no trace of a cellular ancestral signature.

## 3. Changing Perspective: Viruses as Major Players in (Marine) Ecosystems

Recent metagenomic and viromic studies have profoundly shifted our understanding of viral abundance, diversity, and functional importance in the world’s oceans. Perhaps most striking is the finding that viruses are not marginal components, but central actors in marine ecosystems—regulating microbial populations, influencing biogeochemical cycles, and carrying genes that shape host metabolism [39].

One study in the Atlantic Ocean, sampling from the surface down to depths of over 4000 m, used viromics and quantitative methods to characterize RNA viruses. This work identified 2481 putative RNA viral contigs (>500 bp) and 107 complete RNA viral genomes (>2.5 kb), many belonging to largely novel clades such as the Yangshan assemblage and *Nodaviridae*. These viruses were found to be highly endemic (only ~15% matched sequences in Tara Oceans metatranscriptomes outside their locality), with virus-like particle counts reaching ~10^6^ VLP/mL in surface and deep chlorophyll maximum zones. The study estimated that RNA viruses may contribute approximately 5.2–24.4% of the total virioplankton biomass in those zones, with DNA viruses remaining numerically dominant (~10^7^ VLP/mL) [40].

Complementing that, investigation into global deep-sea sediments (133 samples from three oceans) revealed 85,059 viral operational taxonomic units (vOTUs), of which only 1.72% corresponded to previously known virus types. Among these, 1463 deep-sea RNA viruses with complete genomes were identified. The differentiation of viral communities was found to be driven primarily by deep-sea ecosystem type rather than geographic region. Further, virus-encoded metabolic genes—particularly involved in energy metabolism—played a major role in structuring community differences [41].

Another seminal global RNA virome study, based on ~28 terabases of ocean RNA sequence data, proposed five novel RNA virus phyla—among them *Taraviricota* and *Arctiviricota*—which were widespread and relatively abundant. These additions imply that the current taxonomy of RNA viruses underestimates both diversity and ecological significance. The findings indicate that these new phyla occupy substantial ecological niches in oceanic waters, potentially acting as harmless forerunners of potentially pathogenic RNA viruses [40].

## 4. Conclusions and Outlook

Any theory of virus origins must account not only for their emergence through exogenization or microbial degeneration but also for their extensive ecological and symbiotic roles. Like bacteria shortly after their 19th-century discovery, most viruses remain largely unseen, with research historically focused on pathogenic species while the vast majority of harmless or beneficial viruses go unnoticed. It is now clear that viruses contribute substantially to marine biomass, drive nutrient and carbon cycling via the “viral shunt” (where viral lysis releases organic matter back into the environment; Wilhelm & Suttle, 1999 [42]), modulate host metabolism through auxiliary metabolic genes, and may shape host genomes via frequent gene exchange.

Recognition that viruses are integral, quantitatively dominant, and functionally active members of marine ecosystems underscores the need to view them not merely as parasites or biologically irrelevant entities, but as dynamic partners that interact with hosts and influence biological processes. At the same time, this vast virosphere represents a reservoir of potential pathogenic viruses, which may become virulent through degenerative processes or via genetic mechanisms such as recombination and the acquisition of virulence factors. Appreciating these ecological and symbiotic dimensions provides essential context and empirical data for evaluating hypotheses about the origin and function of viruses.

## Data Availability

No new data were created or analyzed in this study.
